# Genomic insights into schizophrenia

**DOI:** 10.1098/rsos.230125

**Published:** 2023-02-22

**Authors:** Michael J. Owen

**Affiliations:** Centre for Neuropsychiatric Genetics and Genomics, Neuroscience and Mental Health Innovation Institute and Division of Psychological Medicine and Clinical Neurosciences, Cardiff University, Cardiff CF10 3AT, UK

**Keywords:** schizophrenia, genomics, neurodevelopment, neurobiology, evolution

## Abstract

Schizophrenia is a common, complex, heterogeneous psychiatric syndrome which can have profound impacts on affected individuals and imposes significant burdens on society. Despite intensive research, it has been challenging to understand basic mechanisms and to identify novel therapeutic targets. Given its high heritability and the complexity and inaccessibility of the human brain, much hope has been invested in the application of genomics as a route to better understanding. This work has identified many common and rare risk alleles and laid the foundations for a new generation of mechanistic studies. Genomics has also thrown new light on the relationship between schizophrenia and other psychiatric disorders and revealed its previously unappreciated aetiological relationship with childhood neurodevelopmental disorders, providing further evidence that it has its origins in disturbances of brain development. In addition, genomic findings suggest that the condition reflects fundamental disturbances in neuronal, and particularly synaptic, function that impact broadly on brain function, rather than being a disorder of specific brain regions and circuits. Finally, genomics has provided a plausible solution to the evolutionary paradox of how the condition persists in the face of high heritability and reduced fecundity.

## 1. Introduction

Schizophrenia is a highly heritable psychiatric syndrome with a lifetime prevalence of around 1% that is diagnosed on the basis of a combination of positive (psychotic), negative and disorganized symptoms [1,2]. It is also frequently accompanied by other features including cognitive impairment, affective symptoms and movement disorders. There is significant heterogeneity in symptoms, mode of onset, course and outcome [2,3]. It lacks clear diagnostic boundaries, and there is phenotypic overlap with other psychiatric disorders, particularly bipolar disorder and childhood neurodevelopmental disorders (NDDs) [4,5]. Current drug treatments largely target only psychotic symptoms, are ineffective in 30% of cases and are associated with adverse effects. Badly needed advances in therapeutics have been impeded by poor understanding of pathophysiology, clinical heterogeneity and a lack of valid biomarkers [2].

Given the high heritability of schizophrenia and the complexity and inaccessibility of the human brain, much has been invested in the application of genomics as a route to better understanding. In this brief perspective I will summarize the considerable progress that has been made over the past 15 years and consider what important insights have come from this research. I will consider four main questions. First, what is the relationship between schizophrenia and other psychiatric disorders? Second, what neurobiological mechanisms are implicated by genetics? Third, when do the critical pathogenic events occur? Fourth, how does schizophrenia persist in the face of high heritability and reduced fecundity?

## 2. The genetic architecture of schizophrenia

Genomic studies have revealed a condition that is highly polygenic, with risk conferred by alleles across the frequency spectrum [6]. To date, genome-wide association studies (GWAS) have identified 287 loci that contain common risk alleles [7], each of small effect, but many more, probably thousands, exist that are collectively responsible for around a third of genetic liability [8,9]. In addition, at least eight rare copy number variants (CNVs) have been identified that confer substantial individual risk [10,11], and most recently, exome sequencing studies have also implicated rare disruptive mutations of large effect [12–18], with 10 genes implicated by this class of mutation at genome-wide significance [16,17].

## 3. The genetic relationship between schizophrenia and other psychiatric disorders

There is moderate to extensive overlap in common risk alleles between schizophrenia and other psychiatric disorders that suggests significant biological pleiotropy [19], though estimates of shared risk might have been inflated by assortative mating [20]. While a degree of pleiotropy is to be expected, given the lack of clear diagnostic boundaries, the genetic correlation between schizophrenia and other psychiatric disorders is typically less than the within-disorder correlation [19]. These findings suggest that, while current diagnostic schemes may not define biologically distinct conditions, they do group cases whose members have more in common with each other than with psychiatric disorders in general.

Common allele pleiotropy is strongest between schizophrenia and bipolar disorder, where the genetic correlation is around 0.7 [21] and substantially higher than can be plausibly attributed to assortative mating [20]. These two conditions share many phenotypic features, can be difficult to distinguish clinically, and the case for regarding them as distinct disorders has long been challenged [4,5].

Recent research has used various analytic approaches to explore genetic overlaps between schizophrenia and bipolar disorder [22,23], and this indicates that clinical features share genetic risk across the two diagnoses and support the hypothesis that the pattern of psychopathology seen in different individuals reflects the confluence of partly orthogonal symptom dimensions and their underlying genetic risk factors [19].

The pattern of pleiotropy seen for rare risk alleles is somewhat different with evidence for shared risk between schizophrenia and childhood onset NDDs such as intellectual disability (ID), autism and attention deficit hyperactivity disorder (ADHD) [24–26]. There is overlap in genes containing rare disruptive mutations [16,25,27], as well as overlap at the level of specific rare risk alleles, both CNVs and rare disruptive mutations [25,28]. Finally, genes implicated by GWAS are enriched for genes associated with rare disruptive mutations in schizophrenia [7,16,17].

There are many important implications of the pleiotropy between psychiatric disorders that have been reviewed elsewhere [19]. Two stand out. First, the current system of diagnostic classification may not be optimal for either basic or clinical research, and we should consider approaches that cut across or divide current diagnostic groups. For example, these might involve stratifying patients based on phenotypic features, or the presence of a particular aetiological factor such as a rare mutation. These studies will require access to genomic data from samples in which the phenotypes have been measured with greater granularity than has hitherto been the norm [29]. A second important consequence of pleiotropy is that researchers should be extremely cautious interpreting mechanistic studies, whether in humans or model systems, that seek to infer causal relationships between possible underlying genetics or neurobiology and specific phenotypic outcomes.

## 4. What neurobiological mechanisms are implicated by genetics?

It is often assumed that the neurobiology of schizophrenia can be localized to disturbances in specific brain regions or circuits. However, the largest GWAS of schizophrenia to date [7] found that genes with high relative expression in most regions of the human brain were enriched for genetic variants associated with schizophrenia. This study also showed an enrichment of common variant associations in genes expressed in both excitatory and inhibitory CNS neurons, and to fundamental biological processes related to neuronal function, in particular gene-sets related to synaptic structure and function [7]. These findings are in accord with the demonstration of synaptic gene-set enrichments in de novo schizophrenia CNVs [30] and a series of subsequent studies indicating similar enrichments in inherited as well as de novo CNVs and rare disruptive mutations in schizophrenia [11,13,16,31,32].

Interpretation of these findings is constrained by current limitations in understanding of the human brain transcriptome and proteome both regionally and developmentally, but as they stand, they pose an alternative to the localizationist view of schizophrenia by suggesting that the condition may reflect fundamental disturbances in neuronal, and particularly synaptic, function that are not confined to a small number of brain regions and circuits. In other words, it may essentially be a disorder of neuronal activity that has widespread impacts on many brain regions and functions. This proposition is not so radical as it might seem when we consider the very diverse psychopathology of schizophrenia, its association with a broad range of cognitive [33], sensory [34] and motor [35] impairments, and the lack of regional specificity in large-scale structural brain imaging studies [36,37].

This is not to claim that individual symptoms, cognitive impairments or other features of schizophrenia are not associated with dysfunction in specific brain regions or circuits. Indeed, this seems very likely to be the case, with the extensive heterogeneity seen among those with schizophrenia likely to reflect regional and circuit-level variability in the impact of these disturbances on neuronal function.

If schizophrenia is essentially a disorder of neuronal function, then it should be possible to gain further mechanistic insights from studies using traditional animal and novel human cellular model systems, and genomic findings, particularly those implicating specific high-risk mutations, offer robust starting points for these [27,31]. Such studies are likely to become a major focus of research efforts over the coming decade. However, researchers aiming to relate neurobiological dysfunction in specific brain regions or circuits to higher level endophenotypic and phenotypic outcomes will need to be extremely careful in drawing causal inferences [19].

## 5. When do the critical pathogenic events occur?

The idea that schizophrenia has its origins in disturbances of neurodevelopment is not new [38,39]. However, the finding of shared risk mutations between schizophrenia and childhood NDDs outlined above has added considerable support to the so-called neurodevelopmental hypothesis of schizophrenia [25]. Evidence for a shared neurodevelopmental aetiology has also accumulated over recent years from findings that many of the environmental risk factors for schizophrenia impact on the developing brain and are shared with childhood NDDs [2].

Interestingly, the enrichment of rare risk mutations is not equal across NDDs, but is greatest in ID, followed respectively by autism, ADHD and schizophrenia [25]. These findings suggest that NDDs rather than being aetiologically discrete entities, are better conceptualized as lying on a neurodevelopmental continuum, with the major clinical conditions reflecting in part the magnitude of the impact on brain development and resulting functional outcomes [25]. Thus, within this continuum, neurodevelopmental conditions occupy a gradient of decreasing neurodevelopmental impact as follows: ID, autism, ADHD and schizophrenia [25].

Schizophrenia had previously been regarded as being distinct nosologically, pathophysiologically and clinically from childhood NDDs. The evidence for genetic overlap between schizophrenia and childhood NDDs and the notion of a neurodevelopmental continuum represent significant challenges to these views. This again points to the need for more transdiagnostic research and has implications for nosology and clinical practice [25]. If, as the evidence suggests, schizophrenia is essentially a NDD, then this might limit our hope of identifying therapeutically tractable mechanisms. However, there are several grounds for optimism. First, given the variability in outcomes of risk mutations, identifying the mechanisms by which sequelae are mitigated or modified might offer important opportunities for early intervention. In this regard, individuals with pathogenic CNVs offer an important high-risk group in which to study longitudinally the precursors and predictors of subsequent psychopathology and to conduct intervention studies [40]. Second, the synaptic, and other fundamental neuronal processes implicated by genomics, not only play important roles in neurodevelopment, but are also key to activities of the mature brain including cognition, learning and memory [31]. These will probably be more amenable to modification than early neurodevelopmental mechanisms. Third, there is considerable variation in course and outcome of schizophrenia. Understanding the genetic and environmental factors that underlie this variation might offer new approaches to treatmenmt [27,41].

## 6. How does schizophrenia persist in the face of high heritability and reduce fecundity?

Schizophrenia is relatively common and highly heritable but is associated with markedly reduced fecundity [42]. Some light has been thrown upon this so-called ‘evolutionary paradox’ [43] by recent genomic studies. The evolutionary explanation for highly penetrant fitness-reducing mutations is likely to be mutation-selection-balance [44] and evidence to support this has come from studies of schizophrenia risk CNVs [45]. Regarding common schizophrenia risk alleles, genomic studies have shown that weak purifying selection is pervasive [46,47] and that, when the effects of background selection are controlled for, there is little evidence for positive selection [47]. These findings, while not excluding a role for balancing selection at some loci, suggest that mutation-selection-drift is a more generally applicable explanation for the presence of common risk alleles in the face of reduced fecundity [44,47].

Taking the evidence together it seems that schizophrenia results, at least in part, from deleterious genetic variants and environmental exposures that impact on brain development. It is not surprising that many factors can disrupt this process given its complexity, and that the cumulative effects of such events can be pathogenic. This is not to say that, as I have argued above, outcomes are immutable. It is likely that humans have evolved many mechanisms to mitigate the effects of adverse events on brain development and that these may be fruitful lines of therapeutic enquiry.

## 7. Conclusion

Schizophrenia genomics represents a work in progress with only about a third of genetic liability accounted for. It seems likely that further progress will be made particularly as whole-genome sequencing technology is applied to sufficiently large samples [48]. While we are some way from being able to pinpoint specific pathogenic mechanisms, much progress has been made and important insights gained. Schizophrenia has been revealed as highly polygenic, with risk conferred by alleles across the frequency spectrum. These findings can now serve as the basis of mechanistic studies in model systems. There are significant genetic overlaps with other psychiatric disorders particularly bipolar disorder and childhood NDDs. This supports and extends existing notions of schizophrenia as a NDD and points to the need for more transdiagnostic research and novel approaches to stratification. Genomic research suggests that schizophrenia may be essentially a disorder of neuronal activity that has widespread impacts on many brain regions and functions, an insight that has many potentially important ramifications and implications for mechanistic studies. Finally, it provides a solution for the evolutionary paradox of how a relatively common and highly heritable condition can persist in the face of reduced fecundity.

## Data Availability

This article has no additional data.
